# Multivariate and network meta-analysis of multiple outcomes and multiple treatments: rationale, concepts, and examples

**DOI:** 10.1136/bmj.j3932

**Published:** 2017-09-13

**Authors:** Richard D Riley, Dan Jackson, Georgia Salanti, Danielle L Burke, Malcolm Price, Jamie Kirkham, Ian R White

**Affiliations:** 1 Research Institute for Primary Care and Health Sciences, Keele University, Staffordshire, UK; 2 MRC Biostatistics Unit, Cambridge, UK; 3 Institute of Social and Preventive Medicine, University of Bern, Switzerland; 4 University of Ioannina School of Medicine, Ioannina, Greece; 5 Institute of Applied Health Research, University of Birmingham, UK; 6 MRC North West Hub for Trials Methodology Research, Department of Biostatistics, University of Liverpool, Liverpool, UK; 7 MRC Clinical Trials Unit at UCL, London, UK

## Abstract

Organisations such as the National Institute for Health and Care Excellence require the synthesis of evidence from existing studies to inform their decisions—for example, about the best available treatments with respect to multiple efficacy and safety outcomes. However, relevant studies may not provide direct evidence about all the treatments or outcomes of interest. Multivariate and network meta-analysis methods provide a framework to address this, using correlated or indirect evidence from such studies alongside any direct evidence. In this article, the authors describe the key concepts and assumptions of these methods, outline how correlated and indirect evidence arises, and illustrate the contribution of such evidence in real clinical examples involving multiple outcomes and multiple treatments

**Summary points**

Meta-analysis methods combine quantitative evidence from related studies to produce results based on a whole body of research
Studies that do not provide direct evidence about a particular outcome or treatment comparison of interest are often discarded from a meta-analysis of that outcome or treatment comparison
Multivariate and network meta-analysis methods simultaneously analyse multiple outcomes and multiple treatments, respectively, which allows more studies to contribute towards each outcome and treatment comparison
Summary results for each outcome now depend on correlated results from other outcomes, and summary results for each treatment comparison now incorporate indirect evidence from related treatment comparisons, in addition to any direct evidence
This often leads to a gain in information, which can be quantified by the “borrowing of strength” statistic, BoS (the percentage reduction in the variance of a summary result that is due to correlated or indirect evidence)
Under a missing at random assumption, a multivariate meta-analysis of multiple outcomes is most beneficial when the outcomes are highly correlated and the percentage of studies with missing outcomes is large
Network meta-analyses gain information through a consistency assumption, which should be evaluated in each network where possible. There is usually low power to detect inconsistency, which arises when effect modifiers are systematically different in the subsets of trials providing direct and indirect evidence
Network meta-analysis allows multiple treatments to be compared and ranked based on their summary results. Focusing on the probability of being ranked first is, however, potentially misleading: a treatment ranked first may also have a high probability of being ranked last, and its benefit over other treatments may be of little clinical value
Novel network meta-analysis methods are emerging to use individual participant data, to evaluate dose, to incorporate “real world” evidence from observational studies, and to relax the consistency assumption by allowing summary inferences while accounting for inconsistency effects

Meta-analysis methods combine quantitative evidence from related studies to produce results based on a whole body of research. As such, meta-analyses are an integral part of evidence based medicine and clinical decision making—for example, to guide which treatment should be recommended for a particular condition. Most meta-analyses are based on combining results (eg, treatment effect estimates) extracted from study publications or obtained directly from study authors. Unfortunately, relevant studies may not evaluate the same sets of treatments and outcomes, which create problems for meta-analysis. For example, in a meta-analysis of 28 trials to compare eight thrombolytic treatments after acute myocardial infarction, it is unrealistic to expect every trial to compare all eight treatments^1^;in fact a different set of treatments was examined in each trial, with the maximum number of trials per treatment only eight.^1^ Similarly, relevant clinical outcomes may not always be available. For example, in a meta-analysis to summarise the prognostic effect of progesterone receptor status in endometrial cancer, four studies provided results for both cancer specific survival and progression-free survival, but other studies provided results for only cancer specific survival (two studies) or progression-free survival (11 studies).^2^

Studies that do not provide direct evidence about a particular outcome or treatment of interest are often excluded from a meta-analysis evaluating that outcome or treatment. This is unwelcome, especially if the participants are otherwise representative of the population, clinical settings, and condition of interest. Research studies require considerable costs and time and involve precious patient involvement, and simply discarding patients could be viewed as research waste.^3^ ^4^ ^5^ Statistical models for multivariate and network meta-analysis address this by simultaneously analysing multiple outcomes and multiple treatments, respectively. This allows more studies to contribute towards each outcome and treatment comparison. Furthermore, in addition to using direct evidence, the summary result for each outcome now depends on correlated results from related outcomes, and the summary result for each treatment comparison now incorporates indirect evidence from related treatment comparisons.^6^ ^7^ The rationale is that by observing the related evidence we learn something about the missing direct evidence of interest and thus gain some information that is otherwise lost; a concept sometimes known statistically as “borrowing strength.”^6^ ^8^

Multivariate and, in particular, network meta-analyses are increasingly prevalent in clinical journals. For example, a review up to April 2015 identified 456 network meta-analyses of randomised trials evaluating at least four different interventions.^9^ Only six of these 456 were published before 2005, and 103 were published in 2014 alone, emphasising a dramatic increase in uptake in the past 10 years (fig 1). *The BMJ* has published more than any other journal (28; 6.1%). Methodology and tutorial articles about network meta-analysis have also increased in number, from fewer than five in 2005 to more than 30 each year since 2012 (fig 1).^10^

**Figure f1:**
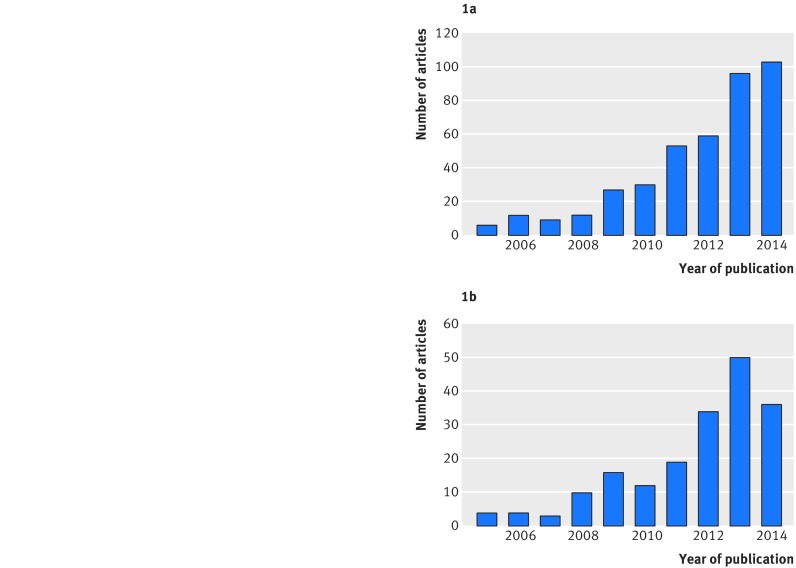
**Fig 1** Publication of network meta-analysis articles over time. (a): Applied articles reporting a systematic reviews using network meta-analysis to compare at least four treatments published between 2005 and 2014, as assessed by Petropoulou et al 2017.^9^ *Six were also published before 2005, and 43 were published in 2015 up to April. (b): Methodological articles, tutorials, and articles with empirical evaluation of methods for network meta-analysis published between 2005 and 2014, as assessed by Efthimiou et al 2016^10^ and available from www.zotero.org/groups/wp4_-_network_meta-analysis

Here we explain the key concepts, methods, and assumptions of multivariate and network meta-analysis, building on previous articles in *The BMJ*.^11^ ^12^ ^13^ We begin by describing the use of correlated effects within a multivariate meta-analysis of multiple outcomes and then consider the use of indirect evidence within a network meta-analysis of multiple treatments. We also highlight two statistics (BoS and E) that summarise the extra information gained, and we consider key assumptions, challenges, and novel extensions. Real examples are embedded throughout.

## Correlated effects and multivariate meta-analysis of multiple outcomes

*Many clinical studies have more than one outcome variable; this is the norm rather than the exception. These variables are seldom independent and so each must carry some information about the others. If we can use this information, we should.*

Bland 2011^14^

Many clinical outcomes are correlated with each other, such as systolic and diastolic blood pressure in patients with hypertension, level of pain and nausea in patients with migraine, and disease-free and overall survival times in patients with cancer. Such correlation at the individual level will lead to correlation between effects at the population (study) level. For example, in a randomised trial of antihypertensive treatment, the estimated treatment effects for systolic and diastolic blood pressure are likely to be highly correlated. Similarly, in a cancer cohort study the estimated prognostic effects of a biomarker are likely to be highly correlated for disease-free survival and overall survival. Correlated effects also arise in many other situations, such as when there are multiple time points (longitudinal data),^15^ multiple biomarkers and genetic factors that are interrelated,^16^ multiple effect sizes corresponding to overlapping sets of adjustment factors,^17^ multiple measures of accuracy or performance (eg, in regard to a diagnostic test or prediction model),^18^ and multiple measures of the same construct (eg, scores from different pain scoring scales, or biomarker values from different laboratory measurement techniques^19^). In this article we broadly refer to these as multiple correlated outcomes.

As Bland notes,^14^ correlation among outcomes is potentially informative and worth using. A multivariate meta-analysis addresses this by analysing all correlated outcomes jointly. This is usually achieved by assuming multivariate normal distributions,^7^ ^20^ and it generalises standard (univariate) meta-analysis methods described previously in *The BMJ*.^12^ Note that the outcomes are not amalgamated into a single outcome; the multivariate approach still produces a distinct summary result for each outcome. However, the correlation among the outcomes is now incorporated and this brings two major advantages compared with a univariate meta-analysis of each outcome separately. Firstly, the incorporation of correlation enables each outcome’s summary result to make use of the data for all outcomes. Secondly, studies that do not report all the outcomes of interest can now be included.^21^ This allows more studies and evidence to be included and consequently can lead to more precise conclusions (narrower confidence intervals). More technical details and software options are provided in supplementary material 1.^22^ ^23^ ^24^ ^25^ We illustrate the key concepts through two examples.

### Example 1: Prognostic effect of progesterone for cancer specific survival in endometrial cancer

In the endometrial cancer example, prognostic results for cancer specific survival are missing in 11 studies (1412 patients) that provide results for progression-free survival. A traditional univariate meta-analysis for cancer specific survival simply discards these 11 studies but they are retained in a multivariate analysis of progression-free survival and cancer specific survival, which uses their strong positive correlation (about 0.8). This leads to important differences in summary results, as shown for cancer specific survival in the forest plot of figure 2. The univariate meta-analysis for cancer specific survival includes just the six studies with direct evidence and gives a summary hazard ratio of 0.61 (95% confidence interval 0.38 to 1.00; I^2^=70%), with the confidence interval just crossing the value of no effect. The multivariate meta-analysis includes 17 studies and gives a summary hazard ratio for cancer specific survival of 0.48 (0.29 to 0.79), with a narrower confidence interval and stronger evidence that progesterone is prognostic for cancer specific survival. The latter result is also more similar to the prognostic effect for progression-free survival (summary hazard ratio 0.43, 95% confidence interval 0.26 to 0.71, from multivariate meta-analysis), as perhaps might be expected.

**Figure f2:**
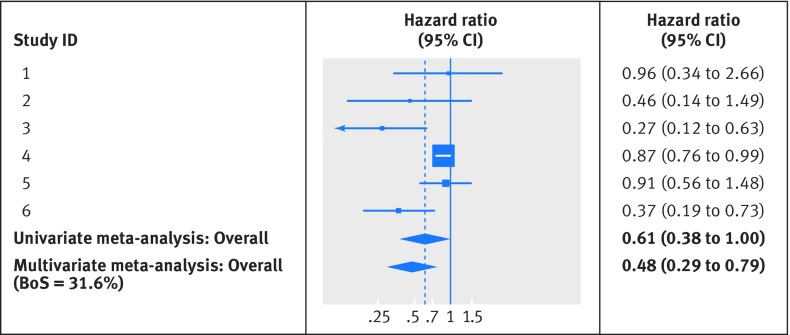
**Fig 2** Forest plot for prognostic effect of progesterone on cancer specific survival in endometrial cancer, with summary results for univariate and multivariate meta-analysis. The multivariate meta-analysis of cancer specific survival and progression-free survival used the approach of Riley et al to handle missing within study correlations, through restricted maximum likelihood estimation.^26^ Heterogeneity was similar in both univariate and multivariate meta-analyses (I^2^=70%)

### Example 2: Plasma fibrinogen concentration as a risk factor for cardiovascular disease

The Fibrinogen Studies Collaboration examine whether plasma fibrinogen concentration is an independent risk factor for cardiovascular disease using data from 31 studies.^17^ All 31 studies allowed a partially adjusted hazard ratio to be obtained, where the hazard ratio for fibrinogen was adjusted for the same core set of known risk factors, including age, smoking, body mass index, and blood pressure. However, a more “fully” adjusted hazard ratio, additionally adjusted for cholesterol level, alcohol consumption, triglyceride levels, and diabetes, was only calculable in 14 studies. When the partially and fully adjusted estimates are plotted in these 14 studies, there is a strong positive correlation (almost 1, ie, a near perfect linear association) between them (fig 3).

**Figure f3:**
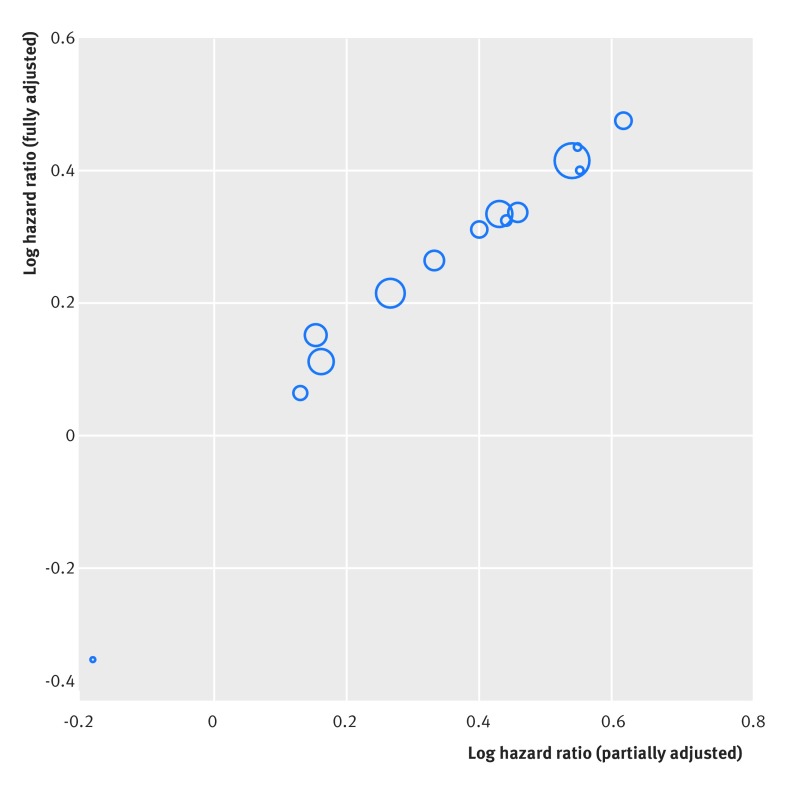
**Fig 3** Strong observed correlation (linear association) between the log hazard ratio estimates of the partially and “fully” adjusted effect of fibrinogen on the rate of cardiovascular disease. The size of each circle is proportional to the precision (inverse of the variance) of the fully adjusted log hazard ratio estimate (ie, larger circles indicate more precise study estimates). Hazard ratios were derived in each study separately from a Cox regression, indicating the effect of a 1 g/L increase in fibrinogen levels on the rate of cardiovascular disease

A standard (univariate) random effects meta-analysis of the direct evidence from 14 trials gives a summary fully adjusted hazard ratio of 1.31 (95% confidence interval 1.22 to 1.42; I^2^=29%), which indicates that a 1 g/L increase in fibrinogen levels is associated, on average, with a 31% relative increase in the hazard of cardiovascular disease. However, a multivariate meta-analysis of partially and fully adjusted results incorporates information from all 31 studies, and thus an additional 17 studies (>70 000 patients), to utilise the large correlation (close to 1). This produces the same fully adjusted summary hazard ratio of 1.31, but gives a more precise confidence interval (1.25 to 1.38) owing to the extra information gained (see forest plot in supplementary material 2).

## Indirect evidence and network meta-analysis of multiple treatments

Let us now consider the evaluation of multiple treatments. A meta-analysis that evaluates a particular treatment comparison (eg, treatment A *v* treatment B) using only direct evidence is known as a pairwise meta-analysis. When the set of treatments differs across trials, this approach may greatly reduce the number of trials for each meta-analysis and makes it hard to formally compare more than two treatments. A network meta-analysis addresses this by synthesising all trials in the same analysis while utilising indirect evidence.^22^ ^27^ ^28^Consider a simple network meta-analysis of three treatments (A, B, and C) evaluated in previous randomised trials. Assume that the relative treatment effect (ie, the treatment contrast) of A versus B is of key interest and that some trials compare treatment A with B directly. However, there are also other trials of treatment A versus C and other trials of treatment B versus C, which provide no direct evidence of the benefit of treatment A versus B, as they did not examine both treatments. Indirect evidence of treatment A versus B can still be obtained from these trials under the so-called “consistency” assumption that, on average across all trials regardless of the treatments compared:

Treatment contrast of A *v* B=(treatment contrast of A *v* C)−(treatment contrast of B *v* C)

where treatment contrast is, for example, a log relative risk, log odds ratio, log hazard ratio, or mean difference. This relation will always hold exactly within any randomised trial where treatments A, B, and C are all examined. It is, however, plausible that it will also hold (on average) across those trials that only compare a reduced set of treatments, if the clinical and methodological characteristics (such as quality, length of follow-up, case mix) are similar in each subset (here, treatment A *v* B, A *v* C, and B *v* C trials). In this situation, the benefit of treatment A versus B can be inferred from the indirect evidence by comparing trials of just treatment A versus C with trials of just treatment B versus C, in addition to the direct evidence coming from trials of treatment A versus B (fig 4).

**Figure f4:**
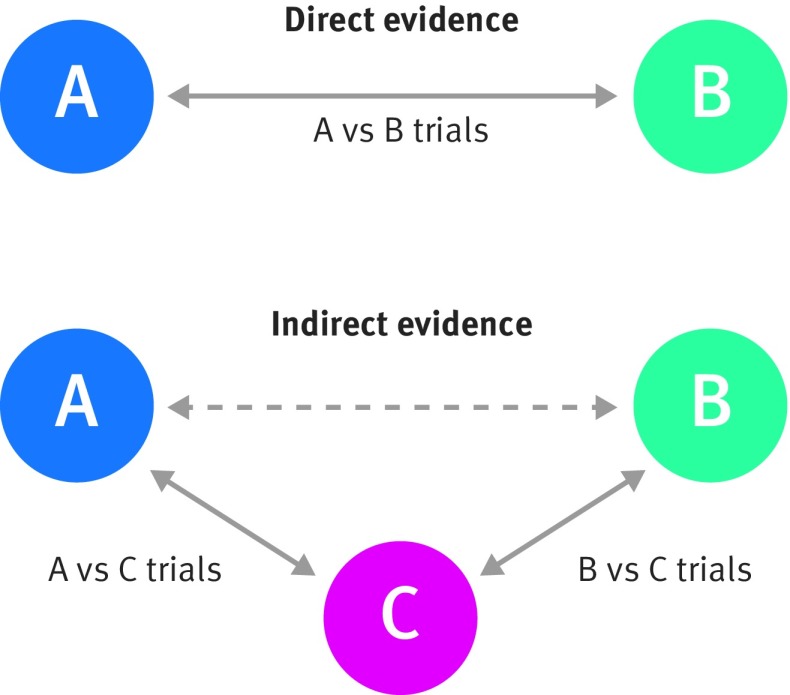
**Fig 4** Visual representation of direct and indirect evidence toward the comparison of A versus B (adapted from Song et al 2011^29^)

Under this consistency assumption there are different options for specifying a network meta-analysis model, depending on the type of data available. If there are only two treatments (ie, one treatment comparison) for each trial, then the simplest approach is a standard meta-regression, which models the treatment effect estimates across trials in relation to a reference treatment. The choice of reference treatment is arbitrary and makes no difference to the results of the meta-analysis. This can be extended to a multivariate meta-regression to accommodate trials with three or more groups (often called multi-arm trials).^30^ ^31^ Rather than modelling treatment effect estimates directly, for a binary outcome it is more common to use a logistic regression framework to model the numbers and events available for each treatment group (arm) directly. Similarly, a linear regression or Poisson regression could be used to directly model continuous outcomes and rates in each group in each trial. When doing so it is important to maintain the randomisation and clustering of patients within trials^30^ and to incorporate random effects to allow for between trial heterogeneity in the magnitude of treatment effects.^12^ Supplementary material 1 gives more technical details (and software options^28^ ^32^) for network meta-analysis, and a fuller statistical explanation is given elsewhere.^30^

After estimation of a network meta-analysis, a summary result is obtained for each treatment relative to the chosen reference treatment. Subsequently, other comparisons (treatment contrasts) are then derived using the consistency relation. For example, if C is the reference treatment in a network meta-analysis of a binary outcome, then the summary log odds ratio (logOR) for treatment A versus B is obtained by the difference in the summary logOR estimate for treatment A versus C and the summary logOR estimate for treatment B versus C. We now illustrate the key concepts through an example.

### Example 3: Comparison of eight thrombolytic treatments after acute myocardial infarction

In the thrombolytics meta-analysis,^1^ the aim was to estimate the relative efficacy of eight competing treatments in reducing the odds of mortality by 30-35 days; these treatments are labelled as A to H for brevity (see figure 5 for full names). A version of this dataset containing seven treatments was previously published in *The BMJ* by Caldwell et al,^13^ and our investigations extend this work.

**Figure f5:**
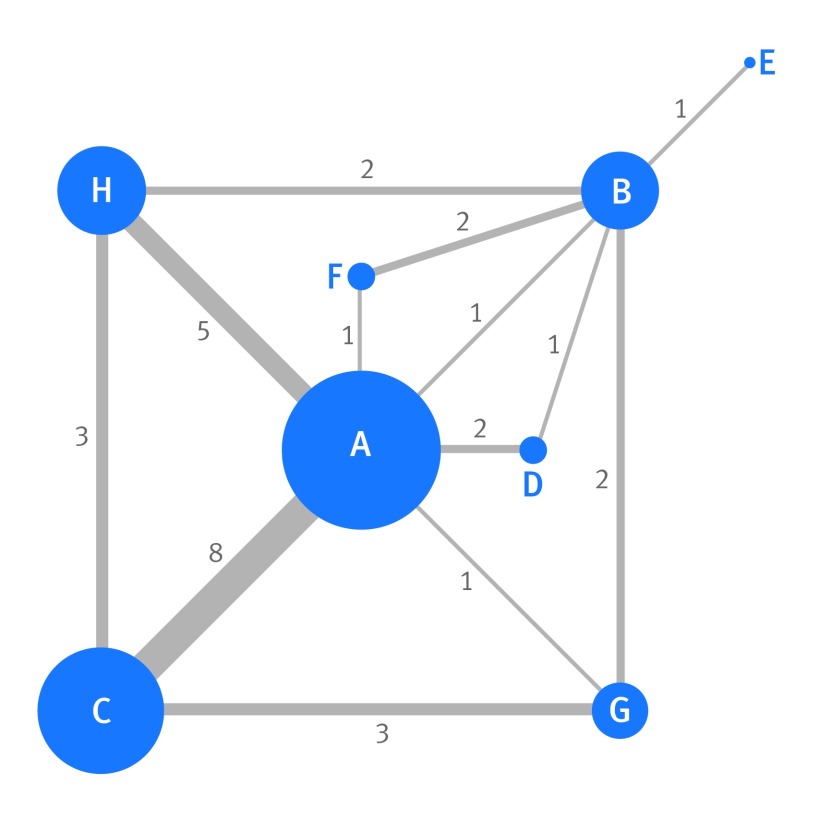
**Fig 5** Network map of the direct comparisons available in the 28 trials examining the effect of eight thrombolytics (labelled A to H) on 30-35 day mortality in patients with acute myocardial infarction. Each node (circle) represents a different treatment, and its size is proportional to the number of trials in which it is directly examined. The width of the line joining two nodes is proportional to the number of trials that directly compare the two respective treatments (the number is also shown next to the line). Where no line directly joins two nodes (eg, treatments C and D), this indicates that no trial directly compared the two respective treatments. A=streptokinase; B=accelerated altepase; C=alteplase; D=streptokinase+alteplase; E=tenecteplase; F=reteplase; G=urokinase; H=anti-streptilase

With eight treatments there are 28 pairwise comparisons of potential interest; however, only 13 of these were directly reported in at least one trial. This is shown by the network of trials (fig 5), where each node is a particular treatment and a line connects two nodes when at least one trial directly compares the two respective treatments. For example, a direct comparison of treatment C versus A is available in eight trials, whereas a direct comparison of treatment F versus A is only available in one trial. With such discrepancy in the amount of direct evidence available for each treatment and between each pair of treatments it is hugely problematic to compare the eight treatments using only standard (univariate) pairwise meta-analysis methods.

Therefore, using the number of patients and deaths by 30-35 days in each treatment group, we applied a network meta-analysis through a multivariate random effects meta-regression model to obtain the summary odds ratios for treatments B to H versus A and subsequently all other contrasts.^28^ ^31^ This allowed all 28 trials to be incorporated and all eight treatments to be compared simultaneously, utilising direct evidence and also indirect evidence propagated through the network via the consistency assumption. The choice of reference group does not change the results, which are displayed in figure 6 and supplementary material 3. The indirect evidence has an important impact on some treatment comparisons. For example, the summary treatment effect of H versus B in the network meta-analysis of all 28 trials (odds ratio 1.19, 95% confidence interval 1.06 to 1.35) is substantially different from a standard pairwise meta-analysis of two trials (summary odds ratio 3.87, 95% confidence interval 1.74 to 8.58).

**Figure f6:**
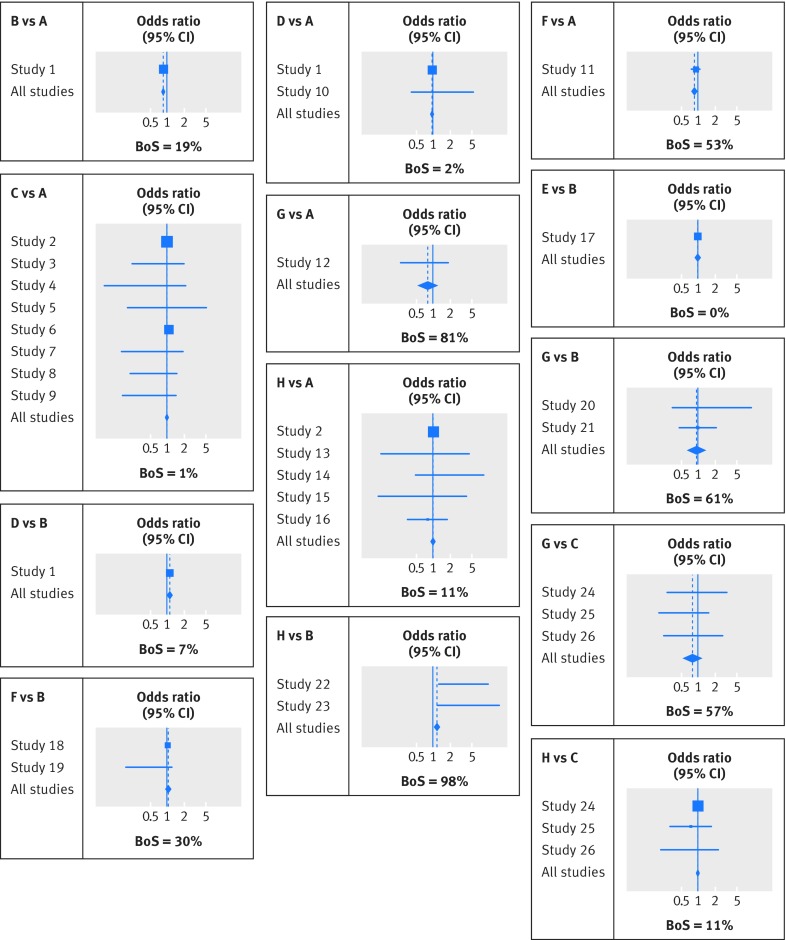
**Fig 6** Extended forest plot showing the network meta-analysis results for all comparisons where direct evidence was available in at least one trial. Each square denotes the odds ratio estimate for that study, with the size of the square proportional to the number of patients in that study, and the corresponding horizontal line denotes the confidence interval. The centre of each diamond denotes the summary odds ratio from the network meta-analysis, and the width of the diamond provides its 95% confidence interval. BoS denotes the borrowing of strength statistic, which can range from 0% to 100%

### Ranking treatments

After a network meta-analysis it is helpful to rank treatments according to their effectiveness. This process usually, although not always,^33^ requires using simulation or resampling methods.^28^ ^31^ ^34^ Such methods use thousands of samples from the (approximate) distribution of summary treatment effects, to identify the percentage of samples (probability) that each treatment has the most (or least) beneficial effect. Panel A in figure 7 shows the probability that each thrombolytic treatment was ranked most effective out of all treatments, and similarly second, third, and so on down to the least effective. Treatment G has the highest probability (51.7%) of being the most effective at reducing the odds of mortality by 30-35 days, followed by treatments E (21.5%) and B (18.3%).

**Figure f7:**
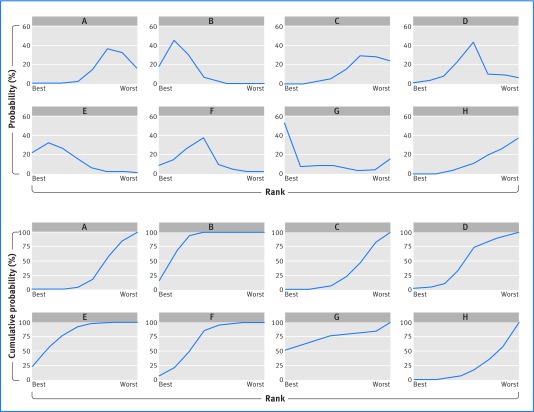
**Fig 7** Plots of the ranking probability for each treatment considered in the thrombolytics network meta-analysis. (Top panel) the probability scale; (bottom panel) the cumulative probability scale

Focusing on the probability of being ranked first is potentially misleading: a treatment ranked first might also have a high probability of being ranked last,^35^ and its benefit over other treatments may be of little clinical value. In our example, treatment G has the highest probability of being most effective, but the summary effect for G is similar to that for treatments B and E, and their difference is unlikely to be clinically important. Furthermore, treatment G is also fourth most likely to be the least effective (14.4%), reflecting a large summary effect with a wide confidence interval. By contrast, treatments B, E, and F have low probability (close to 0%) of being least effective. Thus, a treatment may have the highest probability of being ranked first, when actually there is no strong evidence (beyond chance) that it is better than other available treatments. To illustrate this further, let us add to the thrombolytics network a hypothetical new drug, called Brexitocin, for which no direct or indirect evidence exists. Given the lack of evidence, Brexitocin essentially has a 50% chance of being the most effective treatment but also a 50% chance of being the least effective.

To help address this, the mean rank and the Surface Under the Cumulative RAnking curve (SUCRA) are useful.^36^ ^37^ The mean rank gives the average ranking place for each treatment. The SUCRA is the area under a line plot of the cumulative probability over ranks (from most effective to least effective) (panel B in fig 7) and is just the mean rank scaled to be between 0 and 1. A similar measure is the P score.^33^ For the thrombolytic network (now excluding Brexitocin), treatments B and E have the best mean ranks (2.3 and 2.6, respectively), followed by treatment G (3.0). Thus, although treatment G had the highest probability of being ranked first, based on the mean rank it is now in third place.

## Quantifying the information gained from correlated or indirect evidence

Copas et al (personal communication, 2017) propose that, in comparison to a multivariate or network meta-analysis with the same magnitude of between trial heterogeneity, a standard (univariate) meta-analysis of just the direct evidence is similar to throwing away 100×(1−E)% of the available studies. The efficiency (E) is defined as the:

E=(variance of the summary result based on direct and related evidence)

÷(variance of the summary result based on only direct evidence)

where related evidence refers to either indirect or correlated evidence (or both) and the variance relates to the original scale of the meta-analysis (so typically the log relative risk, log odds ratio, log hazard ratio, or mean difference). For example, if E=0.9 then a standard meta-analysis is similar to throwing away 10% of available studies and patients (and events).

Let us also define n as the number of available studies with direct evidence (ie, those that would contribute towards a standard meta-analysis). Then, the extra information gained towards a particular summary meta-analysis result by using indirect or correlated evidence can be expressed as having found direct evidence from a specific number of extra studies of a similar size to the n trials (see equation 1 in figure 8). For example, if there are nine studies providing direct evidence about an outcome for a standard univariate meta-analysis and E=0.9, then the advantage of using a multivariate meta-analysis is similar to finding direct evidence for that outcome from one further study (see equation 2 in figure 8 for derivation). We thus gain the considerable time, effort, and money invested in about one research study.

**Figure f8:**
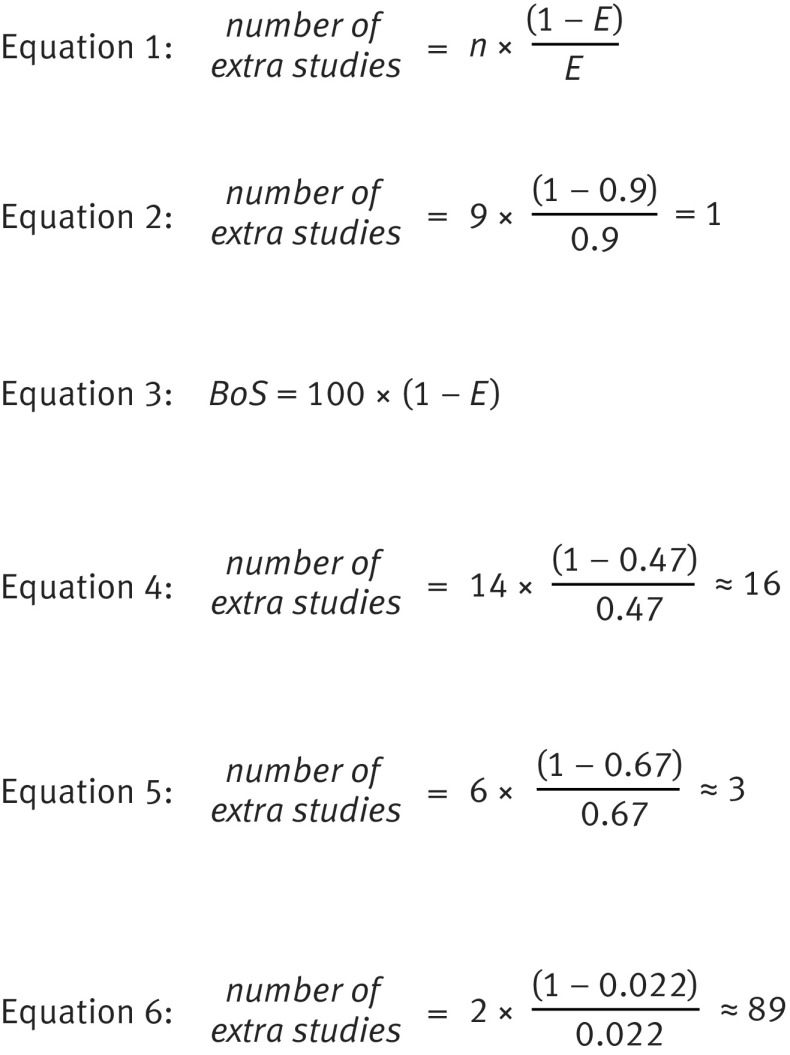
**Fig 8** Equations used to produce figures in the text

Jackson et al also propose the borrowing of strength (BoS) statistic,^8^ which can be calculated for each summary result within a multivariate or network meta-analysis (see equation 3 in figure 8).

BoS provides the percentage reduction in the variance of a summary result that arises from (is borrowed from) correlated or indirect evidence. An equivalent way of interpreting BoS is the percentage weight in the meta-analysis that is given to the correlated or indirect evidence.^8^ For example, in a network meta-analysis, a BoS of 0% indicates that the summary result is based only on direct evidence, whereas a BoS of 100% indicates that it is based entirely on indirect evidence. Riley et al show how to derive percentage study weights for multi-parameter meta-analysis models, including network and multivariate meta-analysis.^38^

### Application to the examples

In the fibrinogen example, the summary fully adjusted hazard ratio has a large BoS of 53%, indicating that the correlated evidence (from the partially adjusted results) contributes 53% of the total weight towards the summary result. The efficiency (E) is 0.47, and thus using the correlated evidence is equivalent to having found fully adjusted results from about 16 additional studies (see equation 4 in figure 8 for derivation).

In the progesterone example, BoS is 33% for cancer specific survival, indicating that using the results for progression-free survival reduces the variance of the summary log hazard ratio for cancer specific survival by 33%. This corresponds to an E of 0.67, and the information gained from the multivariate meta-analysis can be considered similar to having found cancer specific survival results from an additional three studies (see equation 5 in figure 8 for derivation).

For the thrombolytics meta-analysis, BoS is shown in figure 6 for each treatment comparison where there was direct evidence for at least one trial. The value is often large. For example, the BoS for treatment H versus B is 97.8%, as there are only two trials with direct evidence. This is similar to finding direct evidence for treatment H versus B from an additional 89 trials (see equation 6 in figure 8 for derivation) of similar size to those existing two trials. BoS is 0% for treatment E versus B, as there was no indirect evidence for this comparison (fig 6). For comparisons not shown in figure 6, such as treatment C versus B, BoS was 100% because there was no direct evidence. Supplementary material 3 shows the percentage weight (contribution) of each study.

## Challenges and assumptions of multivariate or network meta-analysis

Our three examples show the potential value of multivariate and network meta-analysis, and other benefits are discussed elsewhere.^15^ ^20^ ^39^ The approaches do, however, have limitations.

### The benefits of a multivariate meta-analysis may be small

multivariate and univariate models generally give similar point estimates, although the multivariate models tend to give more precise estimates. It is unclear, however, how often this added precision will qualitatively change conclusions of systematic reviews

Trikalinos et al 2014^40^

This argument, based on empirical evidence,^40^ might be levelled at the fibrinogen example. Although there was considerable gain in precision from using multivariate meta-analysis (BoS=53%), fibrinogen was clearly identified as a risk factor for cardiovascular disease in both univariate and multivariate analyses, and thus conclusions did not change. A counterview is that this in itself is useful to know.

The potential importance of a multivariate meta-analysis of multiple outcomes is greatest when BoS and E are large, which is more likely when:

- the proportion of studies without direct evidence for an outcome of interest is large
- results for other outcomes are available in studies where an outcome of interest is not reported
- the magnitude of correlation among outcomes is large (eg, >0.5 or < −0.5), either within studies or between studies.

In our experience, BoS and E are usually greatest in a network meta-analysis of multiple treatments—that is, more information is usually gained about multiple treatments through the consistency assumption than is gained about multiple outcomes through correlation. A multivariate meta-analysis of multiple outcomes is best reserved for a set of highly correlated outcomes, as otherwise BoS and E are usually small. Such outcomes should be identified and specified in advance of analysis, such as using clinical judgment and statistical knowledge, so as to avoid data dredging across different sets of outcomes. A multivariate meta-analysis of multiple outcomes is also best reserved for a situation with missing outcomes (at the study level), as anecdotal evidence suggests that BoS for an outcome is approximately bounded by the percentage of missing data for that outcome. For example, in the fibrinogen example the percentage of trials with a missing fully adjusted outcome is 55% (=100%×17/31), and thus the multivariate approach is flagged as worthwhile as BoS could be as high as 55% for the fully adjusted pooled result. As discussed, the actual BoS was 53% and thus close to 55%, owing to the near perfect correlation between partially and fully adjusted effects. In contrast, in situations with complete data or a low percentage of missing outcomes, BoS (and thus a multivariate meta-analysis) is unlikely to be important. Also, multivariate meta-analysis cannot handle trials that do not report any of the outcomes of interest. Therefore, although multivariate meta-analysis can reduce the impact of selective outcome reporting in published trials, it cannot reduce the impact of non-publication of entire trials (publication bias).

If a formal comparison of correlated outcomes is of interest (eg, to estimate the difference between the treatment effects on systolic and diastolic blood pressure), then this should always be done in a multivariate framework regardless of the amount of missing data in order to account for correlations between outcomes and thus avoid erroneous confidence intervals and P values.^41^ Similarly, a network meta-analysis of multiple treatments is preferable even if all trials examine all treatments, as a single analysis framework is required for estimating and comparing the effects of each treatment.

### Model specification and estimation is non-trivial

Even when BoS is anticipated to be large, challenges might remain.^20^ Multivariate and network meta-analysis models are often complex, and achieving convergence (ie, reliable parameter estimates) may require simplification (eg, common between study variance terms for each treatment contrast, multivariate normality assumption), which may be open to debate.^20^ ^42^ ^43^ For example, in a multivariate meta-analysis of multiple outcomes, problems of convergence and estimation increase as the number of outcomes (and hence unknown parameters) increase, and so applications beyond two or three outcomes are rare. Specifically, unless individual participant data are available^44^ there can be problems obtaining and estimating correlations among outcomes^45^ ^46^; possible solutions include a bayesian framework utilising prior distributions for unknown parameters to bring in external information.^47^ ^48^ ^49^

### Benefits arise under assumptions

But borrowing strength builds weakness. It builds weakness in the borrower because it reinforces dependence on external factors to get things done

Covey 2008^50^

This quote relates to qualities needed for an effective leader, but it is pertinent here as well. The benefits of multivariate and network meta-analysis depend on missing study results being missing at random.^51^ We are assuming that the relations that we do observe in some trials are transferable to other trials where they are unobserved. For example, in a multivariate meta-analysis of multiple outcomes the observed linear association (correlation) of effects for pairs of outcomes (both within studies and between studies) is assumed to be transferable to other studies where only one of the outcomes is available. This relation is also used to justify surrogate outcomes^52^ but often receives criticism and debate therein.^53^ Missing not at random may be more appropriate when results are missing owing to selective outcome reporting^54^ or to selective choice of analyses.^55^ A multivariate approach may still reduce selective reporting biases in this situation,^39^ although not completely.

In a network meta-analysis of multiple treatment comparisons, the missingness assumption is also known as transitivity^56^ ^57^; it implies that the relative effects of three or more treatments observed directly in some trials would be the same in other trials where they are unobserved. Based on this, the consistency assumption then holds. When the direct and indirect evidence disagree, this is known as inconsistency (incoherence). A recent review by Veroniki et al found that about one in eight network meta-analyses show inconsistency as a whole,^58^ similar to an earlier review.^29^

## How do we examine inconsistency between direct and indirect evidence?

Treatment effect modifiers relate to methodological or clinical characteristics of the trials that influence the magnitude of treatment effects, and these may include length of follow-up, outcome definitions, study quality (risk of bias), analysis and reporting standards (including risk of selective reporting), and the patient level characteristics.^29^ ^59^ ^60^ ^61^ When such effect modifiers are systematically different in the subsets of trials providing direct and indirect evidence, this causes genuine inconsistency. Thus, before undertaking a network meta-analysis it is important to select only those trials relevant for the population of clinical interest and then to identify any systematic differences in those trials providing different comparisons. For example, in the thrombolytics network, are trials of treatment A versus C and treatment A versus H systematically different from trials of treatment C versus H in terms of potential effect modifiers?^62^ If so, inconsistency is likely and so a network meta-analysis approach is best avoided.

It may be difficult to gauge the potential for inconsistency in advance of a meta-analysis. Therefore, after any network meta-analysis the potential for inconsistency should be examined statistically, although unfortunately this is often not done.^63^ The consistency assumption can be examined for each treatment comparison where there is direct and indirect evidence (seen as a closed loop within the network plot)^58^ ^64^ ^65^: here the approach of separating indirect from direct evidence^65^ (sometimes called node splitting or side splitting) involves estimating the direct and indirect evidence and comparing the two. The consistency assumption can also be examined across the whole network using design-by-treatment interaction models,^31^ ^66^ which allow an overall significance test for inconsistency. If evidence of inconsistency is found, explanations should be sought—for example, whether inconsistency arises from particular studies with a different design or those at a higher risk of bias.^56^ The network models could then be extended to include suitable explanatory covariates or reduced to exclude certain studies.^62^ If inconsistency remains unexplained, then the inconsistency terms may instead be modelled as random effects with mean zero, thus enabling overall summary estimates allowing for unexplained inconsistency.^67^ ^68^ ^69^ Other approaches for modelling inconsistency have been proposed,^64^ and we anticipate further developments in this area over the coming years. Often, however, power is too low to detect genuine inconsistency.^70^

In the thrombolytics example, the separating indirect from direct evidence approach found no significant inconsistency except for treatment H versus B, visible in figure 6 as the discrepancy between study 22, study 23, and all studies under the subheading “H *v* B”. However, when we applied the design-by-treatment interaction model there was no evidence of overall inconsistency. If the treatment H versus B studies differed in design from the other studies then it might be reasonable to exclude them from the network, but otherwise an overall inconsistency model (with inconsistency terms included as random effects) may provide the best treatment comparisons.

## Novel extensions and hot topics

### Incorporation of both multiple treatments and multiple outcomes

Previous examples considered either multiple outcomes or multiple treatments. However, interest is growing in accommodating both together to help identify the best treatment across multiple clinically relevant outcomes.^71^ ^72^ ^73^ ^74^ ^75^ ^76^ This is achievable but challenging owing to the extra complexity of the statistical models required. For example, Efthimiou et al^72^ performed a network meta-analysis of 68 studies comparing 13 active anti-manic drugs and placebo for acute mania. Two primary outcomes of interest were efficacy (defined as the proportion of patients with at least a 50% reduction in manic symptoms from baseline to week 3) and acceptability (defined as the proportion of patients with treatment discontinuation before three weeks). These are likely to be negatively correlated (as patients often discontinue treatment owing to lack of efficacy), so the authors extended a network meta-analysis framework to jointly analyse these outcomes and account for their correlation (estimated to be about −0.5). This is especially important as 19 of the 68 studies provided data on only one of the two outcomes. Compared with considering each outcome separately, this approach produces narrower confidence intervals for summary treatment effects and has an impact on the relative ranking of some of the treatments (see supplementary material 4). In particular, carbamazepine ranks as the most effective treatment in terms of response when considering outcomes separately, but falls to fourth place when accounting for correlation.

### Accounting for dose and class

Standard network meta-analysis makes no allowance for similarities between treatments. When some treatments represent different doses of the same drug, network meta-analysis models may be extended to incorporate sensible dose-response relations.^77^ Similarly, when the treatments can be grouped into multiple classes, network meta-analysis models may be extended to allow treatments in the same class to have more similar effects than treatments in different classes.^78^

### Use of individual participant data

Network meta-analysis using aggregate (published) data is convenient, but sometimes published reports are inadequate for this purpose—for example, if outcome measures are differently defined or if interest lies in treatment effects within subgroups. In these cases it may be valuable to collect individual participant data.^79^ As such, methods for network meta-analysis of individual participant data are emerging.^60^ ^80^ ^81^ ^82^ ^83^ ^84^ ^85^ A major advantage is that these allow the inclusion of covariates at participant level, which is important if these are effect modifiers that would otherwise cause inconsistency in the network.

### Inclusion of real world evidence

Interest is growing in using real world evidence from non-randomised studies in order to corroborate findings from randomised trials and to increase the evidence being used towards decision making. Network meta-analysis methods are thus being extended for this purpose,^86^ and a recent overview is given by Efthimiou et al,^87^ who emphasise the importance of ensuring compatibility of the different pieces of evidence for each treatment comparison.

### Cumulative network meta-analysis

Créquit et al^88^ show that the amount of randomised evidence covered by existing systematic reviews of competing second line treatments for advanced non-small cell lung cancer was always substantially incomplete, with 40% or more of treatments, treatment comparisons, and trials missing. To address this, they recommend a new paradigm “by switching: from a series of standard meta-analyses focused on specific treatments (many treatments being not considered) to a single network meta-analysis covering all treatments; and from meta-analyses performed at a given time and frequently out-of-date to a cumulative network meta-analysis systematically updated as soon as the results of a new trial become available.” The latter is referred to as a live cumulative network meta-analysis, and the various steps, advantages, and challenges of this approach warrant further consideration.^88^ A similar concept is the Framework for Adaptive MEta-analysis (FAME), which requires knowledge of ongoing trials and suggests timing meta-analysis updates to coincide with new publications.^89^

### Quality assessment and reporting

Finally, we encourage quality assessment of network meta-analysis according to the guidelines of Salanti et al^90^ and clear reporting of results using the PRISMA-NMA guidelines.^91^ The latter may be enhanced by the presentation of percentage study weights according to recent proposals,^8^ ^38^ to reveal the contribution of each study towards the summary treatment effects.

### Conclusions

Statistical methods for multivariate and network meta-analysis use correlated and indirect evidence alongside direct evidence, and here we have highlighted their advantages and challenges. Table 1 summarises the rationale, benefits, and potential pitfalls of the two approaches. Core outcome sets and data sharing will hopefully reduce the problem of missing direct evidence,^61^ ^79^ ^92^ but are unlikely to resolve it completely. Thus, to combine indirect and direct evidence in a coherent framework, we expect applications of, and methodology for, multivariate and network meta-analysis to continue to grow in the coming years.^9^ ^93^

**Table 1 tbl1:** Summary of multivariate and network meta-analysis approaches

Question	Multivariate meta-analysis of multiple outcomes	Network meta-analysis of multiple treatment comparisons
What is the context?	Primary research studies report different outcomes, and thus a separate meta-analysis for each outcome will utilise different studies	Randomised trials evaluate different sets of treatments, and thus a separate (pairwise) meta-analysis for each treatment comparison (contrast) will utilise different studies
What is the rationale for the method?	• To allow all outcomes and studies to be jointly synthesised in a single meta-analysis model • To account for the correlation among outcomes to gain more information	• To enable all treatments and studies to be jointly synthesised in a single meta-analysis model • To allow indirect evidence (eg, about treatment A *v* B from trials of treatment A *v* C and B *v* C) to be incorporated
What are the benefits of the method?	• Accounting for correlation enables the meta-analysis result of each outcome to utilise the data for all outcomes • This usually leads to more precise conclusions (narrower confidence intervals) • It may reduce the impact of selective outcome reporting	• It provides a coherent meta-analysis framework for summarising and comparing (ranking) the effects of all treatments simultaneously • The incorporation of indirect evidence often leads to substantially more precise summary results (narrower confidence intervals) for each treatment comparison
When should the method be considered?	• When multiple correlated outcomes are of interest, with large correlation among them (eg, > 0.5 or < −0.5) and a high percentage of trials with missing outcomes; or • When a formal comparison of the effects on different outcomes is needed	• When a formal comparison of the effects of multiple treatments is required • When recommendations are needed about the best (or few best) treatments
What are the potential pitfalls of the method?	• Obtaining and estimating within study and between study correlations is often difficult • The information gained by utilising correlation is often small and may not change clinical conclusions • The method assumes outcomes are missing at random, which may not hold when there is selective outcome reporting • Simplifying assumptions may be needed to deal with a large number of unknown variance parameters	• Indirect evidence arises through a consistency assumption—ie, the relative effects of ≥3 treatments observed directly in some trials are (on average) the same in other trials where they are unobserved. This assumption should be checked but there is usually low power to detect inconsistency • Ranking treatments can be misleading owing to imprecise summary results—eg, a treatment ranked first may also have a high probability of being ranked last • Simplifying assumptions may be needed to deal with a large number of unknown variance parameters

## Web extra

Extra material supplied by authors

## Notes

We thank the editors and two reviewers for their constructive comments to improve the article.

Contributors: RDR conceived the article content and structure, with initial feedback from IRW. RDR applied the methods to the examples, with underlying data and software provided by IRW, DJ, and GS. RDR wrote the first draft. All authors helped revise the paper, which included adding new text pertinent to their expertise and refining the examples for the intended audience. RDR revised the article, with further feedback from all other authors. RDR is the guarantor.

Provenance and peer review: Not commissioned; externally peer reviewed.
