# Supplementary material for: Multivariate and network meta-analysis of multiple outcomes and multiple treatments: rationale, concepts, and examples
Source: BMJ. 2017 Sep 13;358:j3932. doi: 10.1136/bmj.j3932 (PMC5596393; doi:10.1136/bmj.j3932)
Supplement: Supplementary file 1 — Supplementary information: additional material [file rilr037955.ww1.pdf]

Supplementary material 1: Technical details of multivariate and network random effects meta-analysis models

Standard (univariate) random effects meta-analysis

A standard random effects meta-analysis combines the study estimates of a single parameter of interest (e.g. a treatment effect) in order to estimate the average parameter value (denoted by μ) and the standard deviation of the parameter values (denoted by τ) across studies. The term 'random' denotes that the true parameter value in an individual study can vary randomly about the average value due to unexplained heterogeneity. If Y_i and var(Y_i) denote the parameter estimate and its variance in study *i*, then assuming within-study and between-study normal distributions, a random effects meta-analysis is: $Y_i \sim N(\mu, \text{var}(Y_i) + \tau^2)$.

Multivariate random effects meta-analysis

A multivariate random effects meta-analysis generalises the standard approach, by allowing for *multiple* correlated parameter estimates to be combined per study, typically by assuming within-study and between-study multivariate normal distributions. For example, in the fibrinogen example a bivariate random effects meta-analysis will jointly synthesise 'fully' adjusted log hazard ratio estimates (Y_{iF}) and partially adjusted estimates (Y_{iP}), whilst accounting for within-study and between-study correlations, to give a summary result for each (μ_F and μ_P, say) and corresponding estimates of between-study standard deviation (τ_F and τ_P). *Within-study correlation* refers to the association between two parameter estimates in the same study, and is caused by the same individuals contributing related data toward each outcome. *Between-study correlation* indicates the strength of association between true parameter values across studies, and is caused by differences across studies in patient and study characteristics modifying the true values in a related way. In the fibrinogen example, high within-study correlation arises due to the same patient data contributing to both 'fully' and partially adjusted estimates, whilst high between-study correlation arises because studies with a higher than average 'fully' adjusted effect will also have a higher than average partially adjusted effect. Between-study correlations are estimated in the meta-analysis, but within-study correlations need to be obtained for each study prior to fitting the meta-analysis. Options have been proposed for dealing with missing within-study correlations, including an alternative approach to multivariate meta-analysis that models an 'overall' correlation, which is an amalgamation of the within and between-study correlations.[1-3]

Network meta-analysis

Network meta-analysis of multiple treatment comparisons can be expressed using various approaches, depending on the data available. If there are only two treatments per trial (i.e. one treatment comparison), then the simplest approach is a standard meta-regression, which regresses the treatment effect estimates against indicator variables that represent particular treatment effects in relation to a chosen reference treatment. This can be extended to a multivariate meta-regression to accommodate trials with multiple treatment comparisons.[4 5] . When the number of events and patients per treatment group are available per study, a hierarchical logistic regression with random effects can be used to model the binomial data directly within trials. Similarly a hierarchical linear regression or Poisson regression with random effects could be used to model continuous outcomes or rates directly. For all approaches, indicator variables are required to model treatment comparisons relative to a particular reference group, and the model framework must preserve the clustering of patients within trials; approaches that ignore this are inappropriate, as they break the original randomisation within each trial.[6-8 7 9] For example, in the logistic regression approach, a separate intercept per trial is needed.

Estimation of the models

We consider the random effects approach more plausible than assuming a fixed (common) effect, and so use random effects models for all our examples. For all the above models, there are many different methods to estimate each μ and τ, and subsequent confidence intervals, including frequentist and Bayesian approaches. In the examples within the paper, we used restricted maximum likelihood (REML) within a frequentist (classical) statistical framework, with confidence intervals accounting for the uncertainty of variance estimates, via the 'mvmeta' and 'network' packages within Stata.[10 11 7 8 12-15] Further, mainly for pragmatic reasons, our network meta-analyses assume a common τ for each treatment comparison. As in a standard meta-analysis, the relative magnitude of heterogeneity can be expressed using multivariate extensions of the I-squared statistic, to give the percentage of the total variability that is due to between-study heterogeneity,[16] and estimates of between-study standard deviation (τ).

An alternative option is a Bayesian approach, which is a natural way to account for all parameter uncertainty, to make predictions and to derive (joint) probabilistic statements regarding the multiple effects of interest. The Bayesian approach combines the likelihood of the observed data with prior probability distributions for the unknown parameters (e.g. treatment effects and between-study variances), to obtain a joint posterior probability distribution of the parameters from which inferences are made. The prior distributions should reflect prior beliefs about possible values of the unknown parameters and can be used to incorporate various sources of uncertainty. It is common for "non-informative" or "flat" prior distributions to be used for unknown parameters, aiming that posterior results should not be influenced by the prior distribution (only the data). However, assessing sensitivity to the choice of such prior distributions is recommended.[17 18] Informative prior distributions based on external (empirical) evidence are also available.[19]

Software

Software for multivariate meta-analysis is available in various statistical packages, including *mvmeta* in Stata[10] and *mvmeta* in R[20], and via PROC MIXED in SAS.[21] Network meta-analysis is often implemented in WinBUGS using, for example, code available at http://www.nicedsu.org.uk/. Dedicated software is also available, such as *network* and *network_graphs* in Stata[11 22] and *netmeta* in R.[23]

Supplementary material 2: Forest plot for the meta-analyses of the ‘fully’ adjusted effect of fibrinogen.

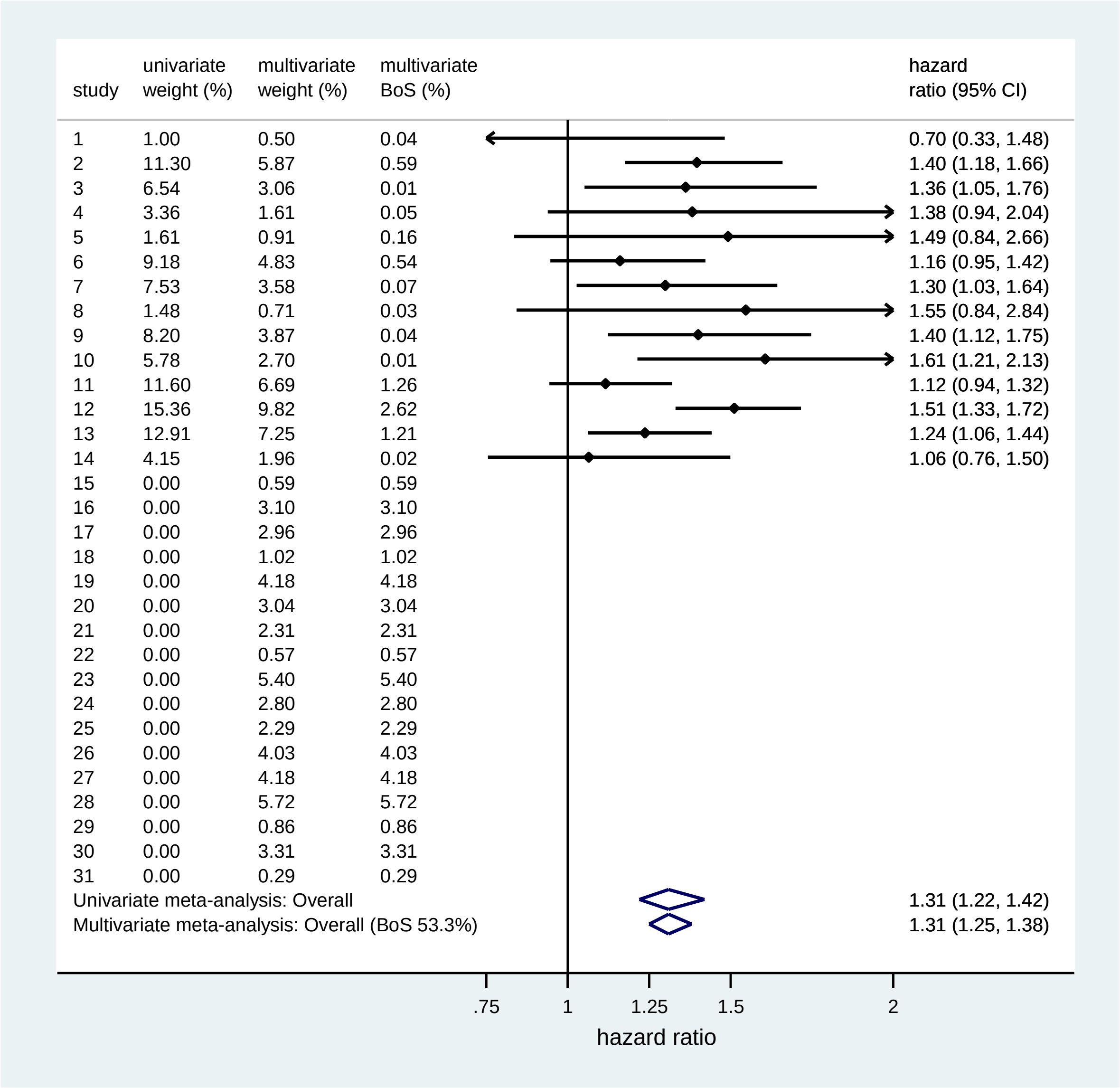

Each solid point denotes the hazard ratio estimate for that study, with its size proportional to the number of patients in that study, and the corresponding horizontal line denotes the confidence interval. The centre of the diamond denotes the summary hazard ratio, and the width of the diamond provides its 95% confidence interval.

Supplementary material 3: Percentage study weights in the network meta-analysis and overall summary results, where treatment A is the reference group; as shown by Riley et al.[24]

	Percentage study weights in the network meta-analysis						
	**B vs A**	**C vs A**	**D vs A**	**E vs A**	**F vs A**	**G vs A**	**H vs A**
**Study 1**	81.14	0.01	97.70	26.67	18.44	0.53	0.01
**Study 2**	0.02	58.05	0	0.01	0	0.23	90.34
**Study 3**	0	0.35	0	0	0	0	0.06
**Study 4**	0	0.14	0	0	0	0	0.02
**Study 5**	0	0.15	0	0	0	0	0.02
**Study 6**	0	39.18	0	0	0	0.15	6.33
**Study 7**	0	0.25	0	0	0	0	0.04
**Study 8**	0	0.43	0	0	0	0	0.07
**Study 9**	0	0.33	0	0	0	0	0.05
**Study 10**	0.05	0	0.55	0.02	0.01	0	0
**Study 11**	10.58	0	0.99	3.48	46.54	0.07	0
**Study 12**	0.13	0.07	0.01	0.04	0.03	19.36	0.01
**Study 13**	0	0.03	0	0	0	0	0.18
**Study 14**	0	0.05	0	0	0	0	0.29
**Study 15**	0	0.03	0	0	0	0	0.18
**Study 16**	0	0.14	0	0	0	0	0.86
**Study 17**	0	0	0	67.13	0	0	0
**Study 18**	5.82	0	0.54	1.91	33.80	0.04	0
**Study 19**	0.12	0	0.01	0.04	0.68	0	0
**Study 20**	0.11	0.03	0.01	0.04	0.03	6.72	0
**Study 21**	0.51	0.12	0.05	0.17	0.12	31.02	0.02
**Study 22**	0.77	0.09	0.07	0.25	0.17	0	0.56
**Study 23**	0.49	0.06	0.05	0.16	0.11	0	0.35
**Study 24**	0.08	0.09	0.01	0.03	0.02	12.07	0.01
**Study 25**	0.11	0.12	0.01	0.04	0.03	17.24	0.02
**Study 26**	0.08	0.09	0.01	0.03	0.02	12.55	0.01
**Study 27**	0	0.14	0	0	0	0	0.36
**Study 28**	0	0.07	0	0	0	0	0.19
**TOTAL**	**100**	**100**	**100**	**100**	**100**	**100**	**100**
**SUMMARY LOG ODDS RATIO (s.e.)**	-0.161 (0.046)	0.002 (0.032)	-0.044 (0.049)	-0.156 (0.080)	-0.113 (0.062)	-0.197 (0.222)	0.014 (0.039)

Supplementary material 4: Ranking of antimanic drugs for response and acceptability, based on either (a) a network meta-analysis of each outcome separately, and (b) a network meta-analysis of both outcomes jointly, accounting for their negative correlation. Treatments located in the darker areas of the plots have the worst rankings, and those in the lighter areas have the best rankings.

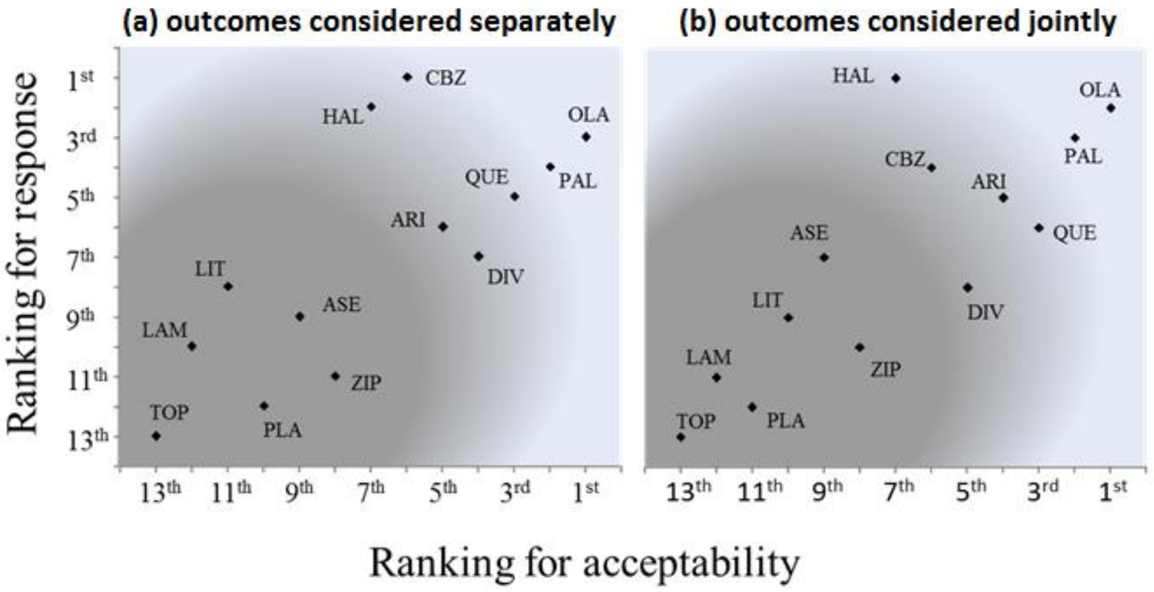

ARI = aripiprazole, ARI = aripiprazole. ASE = asenapine. CBZ = carbamazepine. VAL = divalproex. HAL = haloperidol. LAM = lamotrigine. LIT = lithium. OLZ = olanzapine, PBO = placebo. QTP = quetiapine. PAL = paliperidone, TOP = topiramate. ZIP = ziprasidone.

Reference List for Supplementary Material

1. Riley RD, Thompson JR, Abrams KR. An alternative model for bivariate random-effects meta-analysis when the within-study correlations are unknown. *Biostatistics* 2008;9(1):172-86.
2. Wei Y, Higgins JP. Estimating within-study covariances in multivariate meta-analysis with multiple outcomes. *Stat Med* 2013;32(7):1191-205.
3. Bujkiewicz S, Thompson JR, Sutton AJ, Cooper NJ, Harrison MJ, Symmons DP, Abrams KR. Multivariate meta-analysis of mixed outcomes: a Bayesian approach. *Stat Med* 2013;32(22):3926-43.
4. Salanti G, Higgins JP, Ades AE, Ioannidis JP. Evaluation of networks of randomized trials. *Stat Methods Med Res* 2008;17(3):279-301.
5. White IR, Barrett JK, Jackson D, Higgins JPT. Consistency and inconsistency in network meta-analysis: model estimation using multivariate meta-regression. *Res Synth Method* 2012;3: :111-25.
6. Dias S, Ades AE. Absolute or relative effects? Arm-based synthesis of trial data. *Res Synth Methods* 2016;7(1):23-8.

7. Bafeta A, Trinquart L, Seror R, Ravaud P. Reporting of results from network meta-analyses: methodological systematic review. *BMJ* 2014;348:g1741.
8. Mills EJ, Thorlund K, Ioannidis JP. Demystifying trial networks and network meta-analysis. *BMJ* 2013;346:f2914.
9. Glenny AM, Altman DG, Song F, Sakarovitch C, Deeks JJ, D'Amico R, Bradburn M, Eastwood AJ, International Stroke Trial Collaborative G. Indirect comparisons of competing interventions. *Health Technol Assess* 2005;9(26):1-134, iii-iv.
10. White IR. Multivariate random-effects meta-regression: Updates to mvmeta. *The STATA Journal* 2011;11:255-70.
11. White IR. Network meta-analysis. *The Stata Journal* 2015;15:951-85.
12. Jackson D, Riley RD. A refined method for multivariate meta-analysis and meta-regression. *Stat Med* 2014;33(33):541-54.
13. Song F, Altman DG, Glenny AM, Deeks JJ. Validity of indirect comparison for estimating efficacy of competing interventions: empirical evidence from published meta-analyses. *BMJ* 2003;326(7387):472.
14. Song F, Loke YK, Walsh T, Glenny AM, Eastwood AJ, Altman DG. Methodological problems in the use of indirect comparisons for evaluating healthcare interventions: survey of published systematic reviews. *BMJ* 2009;338:b1147.
15. Song F, Xiong T, Parekh-Bhurke S, Loke YK, Sutton AJ, Eastwood AJ, Holland R, Chen YF, Glenny AM, Deeks JJ, Altman DG. Inconsistency between direct and indirect comparisons of competing interventions: meta-epidemiological study. *BMJ* 2011;343:d4909.
16. Jackson D, White IR, Riley RD. Quantifying the impact of between-study heterogeneity in multivariate meta-analyses. *Stat Med* 2012;31(29):3805-20.
17. Lambert PC, Sutton AJ, Burton PR, Abrams KR, Jones DR. How Vague is Vague? A simulation study of the impact of the use of vague prior distributions in MCMC. *Stat Med* 2005;24:2401-28.
18. Burke DL, Bujkiewicz S, Riley RD. Bayesian bivariate meta-analysis of correlated effects: Impact of the prior distributions on the between-study correlation, borrowing of strength, and joint inferences. *Stat Methods Med Res* 2016.
19. Turner RM, Davey J, Clarke MJ, Thompson SG, Higgins JP. Predicting the extent of heterogeneity in meta-analysis, using empirical data from the Cochrane Database of Systematic Reviews. *Int J Epidemiol* 2012;41(3):818-27.
20. Gasparrini A, Armstrong B, Kenward MG. Multivariate meta-analysis for non-linear and other multi-parameter associations. *Stat Med* 2012;31:3821-39.
21. Van Houwelingen HC, Arends LR, Stijnen T. Advanced methods in meta-analysis: multivariate approach and meta-regression. *Stat Med* 2002;21(4):589-624.
22. Chaimani A, Salanti G. Visualizing assumptions and results in network meta-analysis: the network graphs package. *Stata Journal* 2015;15:905-50.
23. Rucker G, Schwarzer G. netmeta: An R package for network meta analysis. *The R Project website* 2013.
24. Riley RD, Ensor J, Jackson D, Burke DL. Deriving percentage study weights in multi-parameter meta-analysis models: with application to meta-regression, network meta-analysis and one-stage individual participant data models. *Stat Methods Med Res* 2017:962280216688033.
